# ^99m^Tc-EDTA-MN的制备及其在荷瘤裸鼠显像中的初步评价

**DOI:** 10.3779/j.issn.1009-3419.2015.07.06

**Published:** 2015-07-20

**Authors:** 永帅 齐, 贵平 李, 晓华 池, 丽 杜, 凯 黄, 辉 张, 宝丹 黄

**Affiliations:** 510515 广州，南方医科大学南方医院核医学科 Department of Nuclear Medicine, Nanfang Hospital, Southern Medical University, Guangzhou 510515, China

**Keywords:** 肺肿瘤, 乏氧显像剂, 乙二胺四乙酸-甲硝唑酯, 放化纯度, Lung neoplasms, Hypoxia imaging agent, Ethylenediaminetetraacetic Acid-metronidazole, Tadiochemical purity

## Abstract

**背景与目的:**

乏氧是实体肿瘤的一个重要生物学特征，组织乏氧导致肿瘤对放疗和化疗不敏感，从而增加其对放化疗的抵抗性，因此确定肿瘤组织乏氧程度具有重要意义。核医学乏氧显像能够反映肿瘤组织乏氧程度，他能选择性地滞留在乏氧细胞或组织中，主要包括硝基咪唑类和非硝基咪唑类；其中硝基咪唑类肿瘤乏氧细胞显像剂是国内外研究的较多并且具有发展前景的一类用于肿瘤治疗的药物。本研究应用放射性核素标记技术对研制具有临床应用价值的肿瘤乏氧显像剂乙二胺四乙酸-甲硝唑酯(ethylenediaminetetraacetic acid-metronidazole, EDTA-MN)配体进行标记。探讨采用氯化亚锡还原法法进行^99m^Tc标记的可行性，评价^99m^Tc-EDTA-MN的放化特性，观察^99m^Tc-EDTA-MN在非小细胞肺癌(non-small cell lung cancer, NSCLC)裸鼠模型体内的生物学分布特点，为其进一步研究与应用提供实验依据。

**方法:**

采用氯化亚锡还原法进行EDTA-MN配体的^99m^Tc标记，分别对反应量(10 mg、5 mg、2 mg)、氯化亚锡用量(8 mg/mL、4 mg/mL、2 mg/mL)、标记体系pH(2、4、5、6)逐一进行试验，采用正交试验设计进行分析，以寻找最佳标记条件。采用纸层析法和高效液相色谱法测定标记率、放化纯度、脂水分配系数及室温下在生理盐水的体外稳定性，并对标记物在裸鼠体内的生物学分布进行初步显像研究。

**结果:**

最佳标记条件下的标记率(84.11±2.83)%，放射化学纯度大于90%，室温下放置12 h在生理盐水中较稳定。脂水分配系数为lg*P*=-3.05。尾静脉注射^99m^Tc-EDTA-MN的3 h内，裸鼠肿瘤模型初步显像示0.5 h时膀胱区域见大量放射性浓聚，1 h时肿瘤显影清晰，其余脏器内放射性分布均较低。采用ROI技术测得0.5 h、1 h、2 h、3 h肿瘤部位单位面积内放射性计数(88.14±11.59)、(123.17±9.06)、(98.08±14.40)和(79.87±10.57)，并计算肿瘤与肝脏区域的T/NT比值分别为(1.95±0.19)、(3.58±0.78)、(3.95±0.39)和(5.01±0.28)。

**结论:**

^99m^Tc-EDTA-MN易于制备具有比较理想的体内分布动力学特性，可满足后续进一步的体内、外实验研究。

## Abstract (en)

**Background and objective:**

Hypoxia is an important biological characteristics of solid tumor, it is not sensitive to radiotherapy and chemotherapy for which is the presence of hypoxic cell, thus increasing their resistance to conventional radiotherapy and chemotherapy, therefore, the detection of hypoxia degree of tumor tissue is of great significance. The hypoxia imaging of nuclear medicine can reflect the degree of tissue hypoxia, which can selectively retained on the hypoxic cells or tissues, including nitroimidazole and non nitroimidazole; the nitroimidazole is widely and deeply researched as hypoxic celles developer in China and abroad at present. The research about application of radionuclide labelled technique has clinical application value to develop the hypoxia imaging agent EDTA-MN complexes which was labeled. To study the feasibility of ^99m^Tc by direct labeling method, the radiochemical properties evaluation of ^99m^Tc-EDTA-MN, and observe the distribution characteristics of ^99m^Tc radiolabeled EDTA-MN in the xenograft lung cancer nude mice bearing non-small cell lung cancer cell (A549), and provide experimental evidence for its further research and application.

**Methods:**

The radiolabeling of EDTA-MN with ^99m^Tc was performed with direct labeling method, respectively, on the reaction dosage (10 mg, 5 mg, 2 mg), stannous chloride dosage (8 mg/mL, 4 mg/mL, 2 mg/mL), mark system pH (2, 4, 5, 6) one by one test, using orthogonal design analysis, to find the optimal labeling conditions. Labelling rate, radiochemical purity, lipid-water partition coefficient and in vitro stability in normal saline (NS) were determined by TLC and HPLC, and the preliminary study on the distribution of ^99m^Tc-EDTA-MN in nude mice.

**Results:**

The labeling rate of ^99m^Tc-EDTA-MN with the best labeling conditions was (84.11±2.83)%, and the radiochemical purity was higher than 90% by HPLC purification, without any notable decomposition at room temperature over a period of 12 h. The partition coefficient was lg*P*=-3.05, indicated that this complex was hydrophilic. At 3 h post-injection, the imaging of ^99m^Tc-EDTA-MN in nude mice bearing non-small cell lung cancer cell showed that more radioactive gathered in bladder at 0.5 h, the transplanted tumor was clearly imaged at 1 h post-injection, during whole imaging radioactive in other tissues and organs was low. The radioactivity of tumor uptake by using of ROI technology were (88.14±11.59), (123.17±9.06), (98.08±14.40) and (79.87±10.57) at 0.5, 1, 2, 3 h post-injection, and the ratio of T/NT of tumor and liver area were (1.95±0.19), (3.58±0.78), (3.95±0.39) and (5.01±0.28), respectively. ^99m^Tc-EDTA-MN could be quickly cleared from the blood in mice primarily through the kidneys, and the radioactivity in other tissues and organs remained low.

**Conclusion:**

^99m^Tc-EDTA-MN can be easily prepared and labeled compound with high labeling rate and stability, it appears to be suitable for further experiments requirement *in vivo* and *in vitro* application.

目前认为在实体肿瘤中，由于肿瘤生长迅速，造成肿瘤细胞往往处于缺氧状态，肿瘤细胞的乏氧程度越高，这些细胞对放疗和化疗的敏感性越差，临床上很难被彻底杀死。乏氧显像作为检测肿瘤组织乏氧程度的一种无创伤性手段，对于最佳治疗方案的选择、监测疗效以及预后评价具有重要意义^[1-5]^。本研究应用放射性核素标记技术对研制具有临床应用价值的肿瘤乏氧显像剂EDTA-MN配体进行标记。^99m^Tc-乙二胺四乙酸-甲硝唑酯(ethylenediaminetetraacetic acid-metronidazole, EDTA-MN)是一种2-硝基咪唑类化合物，其中EDTA是一种常见且易得的络合剂，为双功能螯合剂；MN是硝基咪唑类化合物，作为靶向基团浓聚于乏氧组织中，其材料来源广泛。

## 1 材料和方法

### 1.1 实验动物

BALB/c裸鼠3只，5周-6周龄，体重20 g左右，SPF级，由南方医科大学实验动物中心提供，动物饲养及实验操作经南方医科大学实验动物伦理委员会批准。

### 1.2 主要试剂和仪器

试剂：广东化工大学轻工业学院惠赠EDTA-MN配体(图 1)；氯化亚锡(SnCl_2_)购于广东光华科技股份有限公司；抗坏血酸(VitC)购于成都市科龙化工试剂厂；高锝酸钠淋洗液来源于原子高科股份有限公司；无水乙腈购于天津市大茂化学试剂厂；以上试剂均为分析纯。聚酰胺膜购于上海摩速科学器材有限公司；Millex®GS 0.22 µm微孔过滤器购于美国Milipore公司。仪器：高效液相系统购于美国Dionex公司；Radio-TLC薄层扫描仪(Bioscan AR-2000)购于美国Bioscan公司；FA604A电子天平(上海精天电子仪器有限公司)；600型恒温水箱(江苏金坛市中大仪器厂)；ZD-3回旋振荡器(天津市欧诺仪器表有限公司)；SN-682型放射免疫γ计数器(上海核福光电仪器有限公司)；Milliennium VG型SPECT显像仪由美国GE公司生产；FJ-391A2型微机放射性活度计由北京核仪器厂生产。

**1:**
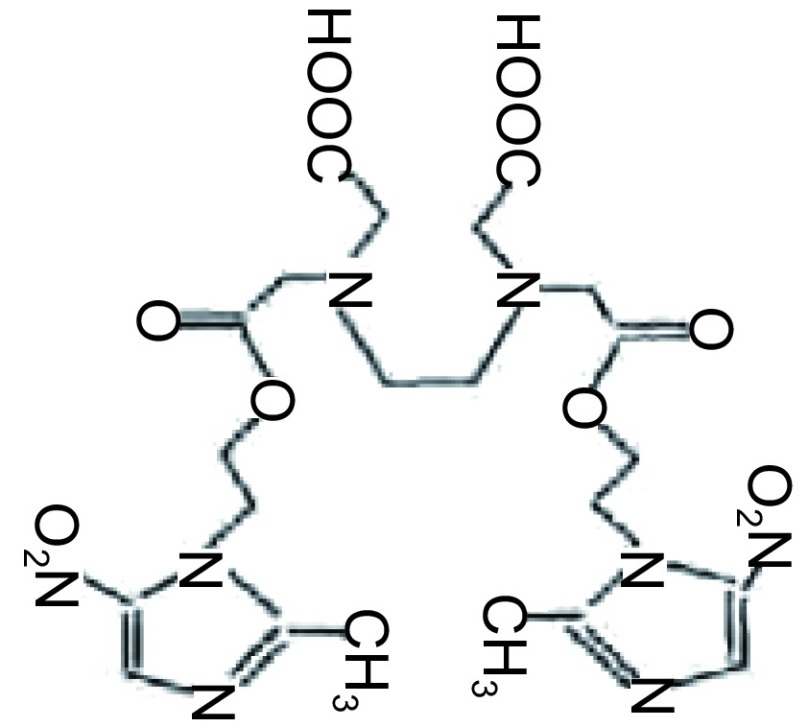
配体EDTA-MN的化学结构 Chemical structures of EDTA-MN

### 1.3 EDTA-MN配体的^99m^Tc标记

采用氯化亚锡还原法法进行EDTA-MN配体的^99m^Tc标记，分别对EDTA-MN配体反应量(10 mg、5 mg、2 mg)、氯化亚锡用量(8 mg/mL、4 mg/mL、2 mg/mL)、标记体系pH(2、4、5、6)逐一进行试验，采用正交试验设计进行分析，以寻找最佳标记条件。将含有一定量EDTA-MN配体的水溶液和新鲜配制0.04 mL SnCl_2_·2H_2_O的HCl溶液(0.02 mol/L HCl溶液)依次加入青霉素瓶，调节溶液pH值，随即加入1 mL高锝酸钠淋洗液(37 MBq)，最后再加入30 mg/mL抗坏血酸0.08 mL溶液，于青霉素瓶置于100 ℃水浴中加热20 min。

### 1.4 ^99m^Tc-EDTA-MN的标记率和放化纯度测定

标记率测定：采用纸层析法，固相为聚酰胺膜，展开剂为60%乙腈水溶液，^99m^TcO_4_^-^、^99m^Tc-胶体、^99m^Tc-EDTA-MN的Rf值分别为0.3-0.5、0.1、0.7-1.0^[6]^。HPLC纯化：采用Kromasil 100-5C18反相柱(250 mm×4.6 mm)，流动相为纯水与甲醇，A相为纯水，B相为甲醇，进样量5 µL，泵流速1 mL/min。

### 1.5 ^99m^Tc-EDTA-MN体外稳定性测定

经HPLC纯化后的^99m^Tc-EDTA-MN分别于室温中放置1 h、3 h、6 h、12 h，然后取少量样品纸层析法测定放化纯度，观察其体外稳定性。

### 1.6 ^99m^Tc-EDTA-MN的脂水分配系数测定

取1 mL 0.02 mol/L磷酸盐缓冲液(pH=7.2)加入10 mL离心试管，再加入1 mL正辛醇和0.01 mL ^99m^Tc-EDTA-MN溶液，盖上塞子，振荡、摇匀，离心5 min(4, 000 r/min)。然后分别从无机相和有机相中取出0.1 mL，测定二相的放射性计数，计算其脂水分配系数*P*(*P*=有机相的放射性活度/无机相的放射性活度)。

### 1.7 荷瘤A549裸鼠模型建立及肿瘤初步显像

取5周-6周龄BALB/c裸鼠3只，体重20 g左右，于右侧腹壁皮下接种A549细胞2×10^6^/只，待肿瘤生长至直径1 cm左右时用于显像。经尾静脉注射标记物4.62 MBq(0.1 mL)，采用低能高分辨准直器，于0.5 h、1 h、2 h、3 h行单光子发射计算机断层成像术(single-photon emission computed tomography, SPECT)平面显像，能峰为140 KeV，矩阵256×256，放大倍数2.0，采集计数700 k左右，初步观察显像剂在肿瘤病灶的浓聚情况，利用感兴趣区(region of interest, ROI)技术，计算肿瘤单位面积内摄取放射性计数以及肿瘤/肝脏区域的放射性计数比值(T/NT)。

## 2 结果

### 2.1 ^99m^Tc-EDTA-MN的最佳标记条件

EDTA-MN用量5 mg、SnCl_2_·2H_2_O用量4 mg/mL、pH值5.0-6.0、反应时间20 min-30 min及反应温度100 ℃为标记物最佳标记条件，该条件下得到标记率为(84.11±2.83)%。

### 2.2 ^99m^Tc-EDTA-MN的标记率和放化纯度测定

最佳标记条件下，^99m^Tc-EDTA-MN的标记率 > 80%，薄层扫描结果如图 2所示。HPLC结果显示制备得到的^99m^Tc-EDTA-MN为一单峰，标准品和标记物在320 nm处紫外吸收峰基本一致，标记物的保留时间约为4 min，放射性化学纯度大于90%(图 3)。

**2:**
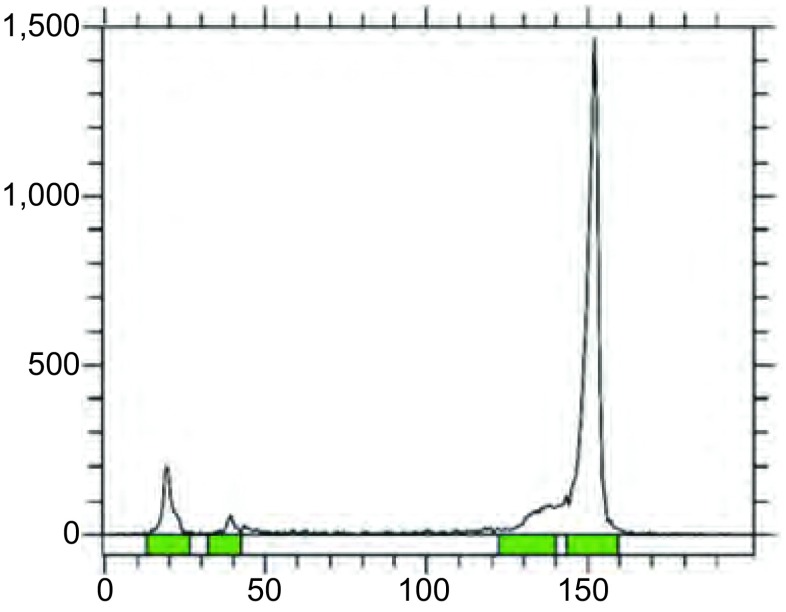
^99m^Tc-EDTA-MN标记率测定的薄层扫描结果 Thin layer chromatography scanning after ^99m^Tc labeling EDTA-MN ligand

**3:**
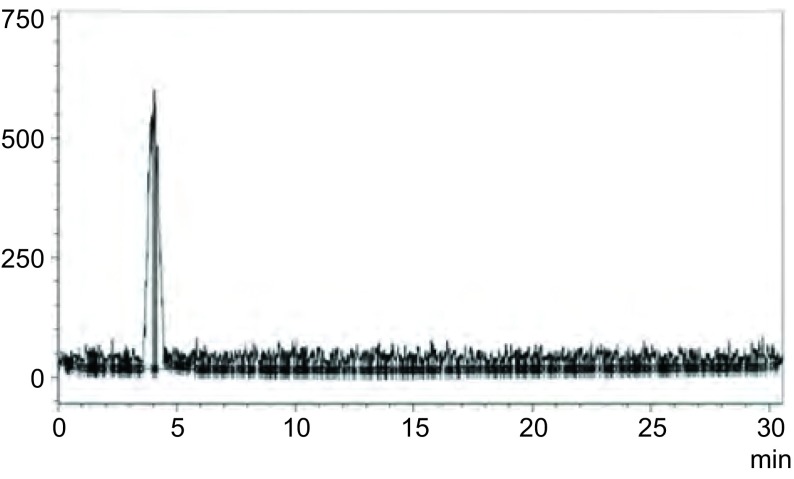
^99m^Tc-EDTA-MN的高效液相色谱图 HPLC chromatogram of ^99m^Tc-EDTA-MN

### 2.2 ^99m^Tc-EDTA-MN体外稳定性测定

室温下在生理盐水中放置1 h、3 h、6 h、12 h后，^99m^Tc-EDTA-MN的放化纯度分别为(93.26±1.10)%、(91.97±0.51)%、(90.15±0.63)%、(88.03±0.11)%。结果表明其放射性化学纯度 > 90%，在室温下标记物具有良好的体外稳定性。

### 2.3 ^99m^Tc-EDTA-MN的脂水分配系数测定

本试验重复3次，根据公式计算得到的平均Log*P*值，试验测得^99m^Tc-EDTA-MN的Lg*P*=-3.05，表明为亲水性更强的物质。

### 2.4 荷瘤A549裸鼠模型建立及肿瘤初步显像

荷瘤裸鼠尾静脉注射标记物后，0.5 h时膀胱区见大量放射性浓聚，肿瘤部位有放射性浓聚，1 h左右即可显像清晰，余器官如脑、肺、肝、脾、肾、胃、小肠及肌肉未见明显放射性浓聚(图 4)。采用ROI技术测得0.5 h、1 h、2 h、3 h肿瘤部位单位面积内放射性计数分别为88.14±11.59、123.17±9.06、98.08±14.40和79.87±10.57，并计算肿瘤与肝脏区域的T/NT比值分别为1.95±0.19、3.58±0.78、3.95±0.39和5.01±0.28。

**4:**
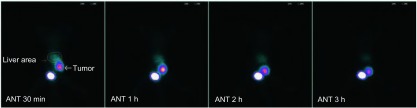
^99m^Tc-EDTA-MN在非小细胞(A549)肺癌裸鼠模型的不同时间显像 ^99m^Tc-EDTA-MN imaging of lung tumor in nude mouse bearing A549 cell at different time points

## 3 讨论

作为肿瘤乏氧显像剂，放射性核素标记的硝基咪唑类化合物目前已得到广泛的研究。全面了解硝基咪唑类乏氧细胞显像剂有效地合成方法，将有助于研究者优化目标配合物的设计和选择高效简洁的合成方式，从而推动先导配合物的发现与结构优化^[5, 7-9]^。放射性核素标记分为直接法和间接法，本研究采用^99m^Tc氯化亚锡还原法与双功能螯合剂EDTA分子络合，利用甲硝唑作为靶向基团浓聚于乏氧组织特点，制备毒性低且稳定性好的^99m^Tc-EDTA-MN乏氧显像剂。在最佳标记条件下，^99m^Tc-EDTA-MN得到标记率达到80%以上，放化纯度大于90%，并且室温下在生理盐水中放置12 h具有良好的体外稳定性。标记物经HPLC分析，EDTA-MN标准品和^99m^Tc-EDTA-MN的主放射峰值时间约为4 min，且在320 nm处紫外检测峰基本一致，表明^99m^Tc已成功标记于EDTA-MN上。

近年来报道的^99m^Tc标记的硝基咪唑类乏氧细胞显像剂普遍存在一些不足：①肿瘤摄取值偏低，显像时间较晚，体内解离快；②肝脏摄取值偏高，肝内活性清除能力差，不利于腹部肿瘤的成像；③肿瘤/血液的比值偏低，药物在血液中清除缓慢。荷瘤裸鼠显像初步结果：30 min时膀胱内可见大量放射性浓聚，1 h左右肿瘤即可显影清晰，3 h时肿瘤部位仍有较多放射性滞留，而脑组织、软组织、甲状腺、胃肠道等部位放射性分布明显降低，表明：①标记物在裸鼠体内血清除较快，亲水性好，主要通过泌尿系统排泄，其余脏器内放射性均较低，有利于减轻对其他器官或组织的辐射损伤；②标记物在体内比较稳定，无明显脱标现象。但肝脏有一过性浓聚，经过肝脏代谢致使^99m^Tc-EDTA-MN在血液中迅速清除，则有利于降低本底，增加靶/非靶组织放射性比值。虽然裸鼠显像示瘤体部位有较高的摄取和很好的滞留，但仍需要进行动物体内分布实验，测定各个脏器的组织重量与其放射性计数得出相应的%ID/g，计算肿瘤摄取值、肿瘤/血液比值、肿瘤/肌肉比值进行实验验证，以寻求高效低毒、稳定性好、结合性强的乙二胺四乙酸-甲硝唑酯乏氧细胞显像剂。

综上所述，放射性核素标记硝基咪唑类化合物作为乏氧细胞显像剂是非常具有应用前景的肿瘤诊断药物。

## References

[1] Jin ZJ, Tang J, Yang Y (2008). The experimental study of ^99m^Tc-EC-MN in tumor hypoxia imaging. Suzhou Da Xue Xue Bao (Yi Xue Ban).

[2] Melo T, Duncan J, Ballinger J R (2000). BRU59-21, a second-generation ^99m^Tc-labeled 2-nitroimidazole for imaging hypoxia in tumors. J Nucl Med.

[3] Giglio J, Fernandez S, Pietzsch HJ (2012). Synthesis, in vitro and in vivo characterization of novel ^99m^Tc-'4+1'-labeled 5-nitroimidazole derivatives as potential agents for imaging hypoxia. Nucl Med Biol.

[4] Huang H, Zhou H, Li Z (2012). Effect of a second nitroimidazole redox centre on the accumulation of a hypoxia marker: synthesis and *in vitro* evaluation of ^99m^Tc-labeled bisnitroimidazole propylene amine oxime complexes. Bioorg Med Chem Lett.

[5] Das T, Banerjee S, Samuel G (2003). ^99m^Tc-labeling studies of a modified metronidazole and its biodistribution in tumor bearing animal models. Nucl Med Biol.

[6] Lin X, Ren JL, Zhang JB (2009). Synthesis and biological evaluation of a novel ^99m^Tc-EDTA-MN complex as a potential agent to target tumor hypoxia. Zhongguo He Ke Xue Ji Shu Jin Zhan Bao Gao.

[7] Mallia MB, Subramanian S, Mathur A (2014). A study on nitroimidazole-^99m^Tc (CO)^3+^ complexes as hypoxia marker: some observations towards possible improvement *in vivo* efficacy. Nucl Med Biol.

[8] Mallia MB, Mathur A, Subramanian S (2005). A novel [^99m^Tc [triple bond]N]^2+^ complex of metronidazole xanthate as a potential agent for targeting hypoxia. Bioorg Med Chem Lett.

[9] Huang J, Feng H, Ma ML (2013). New progress of chemical structure and structure-activity relationship on nitroimidazole as hypoxic celles developer. Xi Hua Da Xue Xue Bao (Zi Ran Ke Xue Ban).

